# Effect of Microgravity Environment on Gut Microbiome and Angiogenesis

**DOI:** 10.3390/life11101008

**Published:** 2021-09-24

**Authors:** Ruqaiyyah Siddiqui, Rizwan Qaisar, Nandu Goswami, Naveed Ahmed Khan, Adel Elmoselhi

**Affiliations:** 1 College of Arts and Sciences, American University of Sharjah, Sharjah 26666, United Arab Emirates; rsiddiqui@aus.edu; 2 College of Medicine, University of Sharjah, Sharjah 27272, United Arab Emirates; rizwan@yahoo.com; 3 Physiology Division, Otto Loewi Research Center for Vascular Biology, Immunology and Inflammation, Medical University of Graz, Neue Stiftingtalstraße 6/D.05, 8010 Graz, Austria; nandu.goswami@medunigraz.at; 4 College of Medicine, Mohammed Bin Rashid University of Medicine and Health Sciences, Dubai 505055, United Arab Emirates

**Keywords:** gut microbiota, microbiome, angiogenesis, microgravity, space travel

## Abstract

Microgravity environments are known to cause a plethora of stressors to astronauts. Recently, it has become apparent that gut microbiome composition of astronauts is altered following space travel, and this is of significance given the important role of the gut microbiome in human health. Other changes observed in astronauts comprise reduced muscle strength and bone fragility, visual impairment, endothelial dysfunction, metabolic changes, behavior changes due to fatigue or stress and effects on mental well-being. However, the effects of microgravity on angiogenesis, as well as the connection with the gut microbiome are incompletely understood. Here, the potential association of angiogenesis with visual impairment, skeletal muscle and gut microbiome is proposed and explored. Furthermore, metabolites that are effectors of angiogenesis are deliberated upon along with their connection with gut bacterial metabolites. Targeting and modulating the gut microbiome may potentially have a profound influence on astronaut health, given its impact on overall human health, which is thus warranted given the likelihood of increased human activity in the solar system, and the determination to travel to Mars in future missions.

## 1. Introduction

The microgravity environment is known to instigate a plethora of stressors to astronauts undertaking space missions [1,2,3]. These stressors can affect gut microbiome composition and may have a negative impact on astronauts’ health. Given that the International Space Exploration Coordination Group, which encompasses 20 national space agencies, issued a third edition of the Global Exploration Roadmap [4] with a strategy to increase human activities in the solar system and the ambitious goal of setting foot on the surface of Mars by 2030, it is imperative that strategies to improve and safeguard astronaut health are devised. Some of the key risks to astronaut health that are associated with space flight and a microgravity environment include musculoskeletal changes such as reduced muscle strength and bone fragility as well the ability of the body to form new capillaries from existing blood vessels, termed “angiogenesis”. Other risks include visual impairment, endothelial dysfunction, metabolic changes, behavior changes due to fatigue or stress and effects on mental well-being, as well as changes due to radiation such as damage to the DNA, as depicted in a recent study conducted by the national aeronautics and space administration (NASA) termed the “NASA twins study”. This study examined the effects of space flight on identical astronaut twins, one who spent almost a year on International Space Station, and the other who was a ground-based control, having remained on earth throughout the duration of the study [5].

Numerous studies have indicated the profound role of the gut microbiome in human health [6,7,8]. The gut microbiota has been shown to influence several areas of human health, such as the immune system and energy metabolism, and is also thought to influence the central nervous system via the neural, endocrine and immune pathways [9] as well as safeguarding against neurodevelopment disorders [3]. Targeting the gut microbiome, with specifically designed probiotics or dietary fiber, which focuses on the issues that are faced by astronauts, could potentially reduce a variety of issues. Supplementation with probiotics has been shown to have beneficial effects on human health. Gut microbiota composition influences response to chemotherapy and immunotherapy as well as blood-glucose response to foods and could be utilized to personalize diet, as studies have shown that people that consume identical meals may depict variable responses to the glucose in their blood, depending on their dietary habits, levels of physical activity as well as their gut microbiome [8,10]. In addition, dietary fiber intake influences gut microbiota composition and is related to better health [11] Studies have revealed that during and after a mission to the International Space Station (ISS), changes in microbial gut diversity were observed in astronauts [12]. Thus, it will be of benefit to modulate the gut microbiome of astronauts in order to improve their overall health, and to understand how the gut microbiome and its metabolites are produced under microgravity conditions, in comparison to normal environments, where it is warranted to protect against any adverse changes visible in astronauts following spaceflight.

A recent study in mice revealed that dysbiosis of the gut leads to elevated intestinal permeability and chronic low-grade inflammation, which eventually causes pathological angiogenesis, which can affect sight, suggesting the role of the gut microbiome in both angiogenesis and vision. The study revealed that a diet that is high in fat can alter the gut microbiome and increase systemic inflammation, heightening pathological choroidal neovascularization [13]. This is of note, as visual impairment is one of the risks that affect astronauts [14,15]. Furthermore, studies have documented a wide spectrum of neuro-ophthalmic alterations in astronauts during and after spaceflight, and this is described by the term Spaceflight Associated Neuro-ocular Syndrome (SANS). Angiogenesis is the formation of new blood vessels from pre-existing vasculature [16] Interestingly, pathologic angiogenesis in the eye can lead to severe visual impairment, pointing to the connection of angiogenesis and visual impairment [17] On the other hand, musculoskeletal changes and reduced muscle strength and bone fragility have also been observed in astronauts and are of concern [18]. Moreover, angiogenesis is known to play a role in both muscle adaptation and the contribution of skeletal muscle development [19] Thus, an understanding of the effects of microgravity on angiogenesis as well as the relationship with the gut microbiome and the resulting effects on astronaut health due to microgravity environments are discussed here. In addition, the connection of angiogenesis with visual impairment, skeletal muscle and gut microbiome is proposed and explored. Furthermore, metabolites as effectors of angiogenesis are deliberated upon and linked with gut bacterial metabolites with a view to understanding their effects and perhaps modulating these in order to improve astronauts’ health.

## 2. Role of Gut Microbiota in Astronaut Health

Many studies suggest that spaceflight has noteworthy effects on gut microbiome composition of astronauts [3,20]. As stated earlier, the gut microbiome is well known to play a fundamental role in the maintenance of the host’s physiological conditions by modulating its immunity. The gut microbiome encompasses an estimated 100 trillion microorganisms (largely bacteria, as well as protozoa, fungi and viruses) and encodes in excess of 3 million genes that can yield thousands of metabolites, with many functions that impact the overall health of the host [8,21]. Multiple reports denote that the microbiome can deliver protection against disorders such as metabolic diseases, inflammatory bowel disease and allergic diseases; while dysbiosis in the gut may in turn result in disease development as well as affecting the immune system [22,23,24]. Gut microbiota may produce short-chain fatty acids which in turn traverse the intestinal epithelia, directly impacting mucosal immune responses [25]. The two noteworthy bacterial species that reside in the gut are the Firmicutes and Bacteroidetes, and a balanced ratio between these species is integral in the maintenance of homeostasis in the host [26]. Firmicutes are gram-positive and are involved in the metabolism and nutrition of the host, regulation of hunger and satiety, via short-chain fatty acid synthesis. By contrast, Bacteroidetes are gram-negative and connected with immunomodulation, and their constituents interact with cell receptors and augment immune reactions through synthesis of cytokines [26]. The gut bacteria and their metabolites may stimulate the enteric nervous system. Moreover, the gut bacteria and metabolites may influence the function of the tissues and organs that control circulatory system homeostasis, for example, the blood vessel wall, blood cells and the heart [27]. The microgravity environment has effects on both the vascular physiology as well as gut microbiome composition, due to the various environmental influences in the space environment, in particular through extended spaceflight travel. In this regard, a large study termed “the Astronaut Microbiome project” is being conducted to study the microbiome of astronauts with an aim to determine microbiome changes during space travel [28]. For example, prolonged exposure to radiation may considerably alter gut microbiome composition and encourage alterations of gut homeostasis. It is hoped that the impact of space travel on gut microbiome of astronauts and their surroundings and the effects on astronaut health will be determined. Currently these data have depicted that astronaut microbiome composition became less diverse [12]. In fact, the study revealed an increase in *Faecalibacterium*, which is beneficial as a butyrate producer. On the other hand, an increase in *Parasutterella* was observed, and these species have been associated with chronic intestinal inflammation. Moreover, decreased amounts of the genera *Akkermansia*, which is associated with anti-inflammatory properties, were observed. Therefore, prebiotics containing *Akkermansia*, which reduce the chance of diseases associated with chronic inflammatory responses, have been suggested [12,20].

Further evidence of the important role the gut microbiome might play has been supplemented by earth-based Analog mission projects, for example, the “MARS500 study”, where six astronauts were confined within an analogue Mars-surface habitat over 520 days [20]. This study examined astronaut gut microbiota over the entire mission, including the period prior to entering confinement and 6 months following the return to normal life. It was revealed that there was an increase in the abundance of *Bacteroides* spp. in the first part of the mission, and decreased abundance of *Faecalibacterium prausnitzii* was observed. These data were similar to those obtained in another study conducted in the 1970s, known as the “Skylab Medical Experiments Altitude Test” in a 56-day confinement environment, which also revealed changes in the ratio of *Bacteroides* to *Firmicutes* [29,30,31]. This was an extensive study, offering an opportunity to investigate the habitability and physiological adaptations to space flight over extended periods of time in the location of a space habitat and an orbital laboratory [29,30,31]. Interestingly, the MARS500 study revealed that the butyrate-producing members of the gut microbiota, namely *F. prausnitzii*, were observed to alter in all the subjects of the study implicating the significance of short-chain fatty acid production, with possible inferences for the maintenance of the microbiome in the subjects. It is worth noting that the study revealed that *Faecalibacterium prausnitzii* reached their lowest values at about 1 year of confinement, with the psychological data suggested that the circumstances may have been stressful and higher intensities of salivary cortisol and increased immune responses were indicative. Nonetheless, other short-chain fatty acid producers, for example *Dorea* were not affected. However, the overall analysis depicted no significant changes overall the astronaut gut microbiome [20].

Recently, the data from the MARS500 project were reevaluated using improved 16S rRNA gene amplicon bioinformatics technology [32]. The fecal samples data from the earlier part of the study (days 7–45) and later part of the study (days 420–520) were re-analyzed, and 408 exact sequence variants (ESVs) were identified. It was found that 32 of the ESVs were significantly and differentially abundant over time. The different ESVs included reduction of keystone resistant starch degrading, insulin sensitivity-associated and anti-inflammatory species, such as *Ruminococcus bromii, Faecalibacterium prausnitzii, Lactobacillus rogosae Anaerostipes hadrus, Blautia luti,* and *Roseburia faecis,* as well as the enrichment of yet-to-be-cultured bacteria. The reanalysis revealed that the gut microbiome was indeed altered in a significant manner throughout the period of the study, comprising species that can induce inflammation as well as glucose homeostasis in their host [32]. Recently some studies have used a shotgun metagenomic sequencing, and microarray approach whereas previous studies utilized 16S rRNA based sequencing [33,34]. The study investigated the microbial profile of the swabs taken of mouth, skin, nose, ear and saliva for an astronaut, both prior to spaceflight as well as during and after return to earth [33]. Moreover, various environmental samples from the ISS habitat were also collected. It was noted that the microbiome of ISS surface environment resembled those of the astronaut’s skin [33]. In another recent study, microbial detection array alongside shotgun metagenomic sequences was accomplished to examine the microbiome of four astronauts, before, during and following spaceflight on the ISS [34]. The study identified that astronaut microbiome composition changed during spaceflight, but returned to normal following return to earth. Furthermore, some of the astronauts showed significant changes in taxonomic abundance as well as diversity where as one astronaut did not. Of note, abundance of *Prevotella* in saliva samples was shown to increase for two astronauts. Interestingly, presence of antimicrobial resistance genes within saliva samples were observed, namely elfamycin resistance gene significantly increased in all four astronauts, but returned to normal following the return to earth [34]. Previously, studies were conducted on *E. coli* in vitro under simulated microgravity conditions [35]. In this study *E. coli* were grown for over 1000 generations and it was found that microgravity-adapted strain readily outcompeted the unadapted strain and genomic sequencing revealed 16 mutations under microgravity conditions. However, the significance changes are not yet known. In this study antibiotic resistance was not reported [35]. Of note, this study was followed up with another study whereby *E. coli* was grown for over 100 generations and resistance to chloramphenicol, cefalotin, cefuroxime axetil, cefoxitin, cefuroxime, and tetracycline was observed with resistance to chloramphenicol and cefalotin persisting for over 110 generations [36].

Alongside studies in astronauts, in vitro and in vivo studies have been conducted in microgravity as well as simulated microgravity environments which portray that the gut microbiome diversity is affected and effects of simulated microgravity on beneficial microbes has been examined [37,38]. It was demonstrated that in the hindlimb-unloading mouse model which mimics microgravity environments, proinflammatory cytokine-mediated intestinal immunity to *Citrobacter rodentium* infection was reduced and disturbance of gut microbiome was induced [37]. Notably, these phenotypes could be largely corrected by introducing a high-concentrated probiotic comprising 8 live freeze-dried bacterial species, indicating its potential as a dietary supplement for astronauts. Future research on bacterial species that induce inflammation such as short-chain fatty acid producers in future missions are warranted, and development of pro/prebiotics based on the species lacking in astronauts should be developed [20]. Further analysis of functional aspects of the microbiome such as investigation of virulence genes, metabolic genes and antibiotic resistance genes will also be necessary to understand the microbial relationship of the environment with astronauts, coupled with future studies on more astronauts to improve statistical significance [39]. In addition, understanding the long-term response of bacteria to microgravity environments is needed.

## 3. Angiogenesis and the Influence of Microgravity

Angiogenesis is the formation of new blood vessels from pre-existing vasculature [16]. Angiogenesis in adults is of importance in processes such as bone repair and wound healing and is regulated by a delicate balance of both pro- and anti-angiogenic molecules. It has been shown in several studies that this balance may be compromised in various diseases such as retinopathy, rheumatoid arthritis, cardiovascular and cerebrovascular disorders and perhaps even tumors and cancer development [27]. Endothelial cells lining the inward surface of vessels play a key aspect in the maintenance of vascular integrity and tissue homeostasis, as they regulate various physiological processes such as blood flow. Thus, endothelial cells are fundamentally involved in aspects of angiogenesis or the formation of new capillaries from existing blood vessels [40]. Various studies have investigated the mechanisms by which microgravity may affect endothelial cell ability to function. Several studies show that endothelial cells are affected by microgravity and go through various biochemical, functional and morphological changes; however, these studies were all mostly conducted under simulated microgravity conditions or on various cell lines in vitro on board the ISS [41,42,43]. This is because there are limited opportunities to conduct experiments on spacecrafts or space laboratories in the real microgravity environment due to high expense and the limited number of missions. Thus, most of these studies were conducted at ‘ground-based facilities’ such as the clinostat or the Random Positioning Machine [44]. However, vascular channel development in the rat fibular osteotomy model was repressed following space travel, implying that bone healing is possibly impaired under microgravity conditions [45]. Another prior study revealed a significant density decrease in small vessels in quail chorioallantoic membranes following space flight [46]. In addition, it was found that orbital spaceflight can reduce wound healing ability in the rat, and angiogenesis plays a crucial part in wound healing [47]. However, despite the limitations, these studies are necessitated as astronauts returning from space travel may have cardiovascular problems such as cardiac atrophy, low blood pressure or orthostatic intolerance [48,49] which may be due to injury in the endothelium [50]. In fact, to date, these studies have revealed that endothelial cells depict alteration in secretory functions as well as the modulation of various processes, namely apoptosis, proliferation, cytoskeletal organization, growth, intracellular signaling mechanisms and growth behavior [44]. Furthermore, augmenting with vascular endothelial growth factor (VEGF) reveals cell-protective effects on the endothelial cells [51]. Further studies are necessary and will aid in the development of possible countermeasures to prevent cardiovascular issues in astronauts.

## 4. Gut Microbiome, Angiogenesis and Link with Visual Impairment

As already stated, the gut microbiome is known to affect a plethora of issues in the host. If there is dysbiosis in the gut microbiota composition, this can affect the intestinal epithelial barrier and challenge the host immune system [3]. Visual impairment called Neuro-ocular Syndrome (SANS) is one of the major problems for astronauts, especially in long-term space flight [52]. It has been reported that 37–51% of astronauts suffer from SANS ocular changes after 6-month missions in a microgravity environment. SANS is described as changes in visual function and ocular structure. The ocular changes of SANS include optic disc edema, choroidal folds, cotton wool spots, retinal nerve fiber layer thickening, decreased near visual acuity, increased optic nerve sheet diameter (ONSD) and eye globe flattening. Microgravity-induced cephalad fluid shift has been reported to be the main cause of SANS, among other factors [15]. However, the exact pathophysiology or mechanism of SANS is not completely understood, and an effective method to countermeasure it is not yet identified. A recent study depicted that simulated microgravity induced significant alterations in cytoskeletal proteins of adult retinal pigment epithelium cells, as well as changes to cell behavior, growth and genetic expression patterns involved in cell structure, growth, shape, migration, adhesion and angiogenesis. This study suggested that there may be weakening of the interior and exterior cellular structures of the retinal cells in a simulated microgravity environment, which might affect the function of the retinal layer. Nonetheless, it was recently reported that human neural stem cells proliferate when in space and express specific markers [53]. Neural stem cells are integral in the maintenance of the central nervous system’s function. In addition, the cells revealed higher oxygen consumption and glycolysis compared with ground controls. These cells also kept their ability to become young neurons [53]. This was further corroborated in another study, which revealed that human neural stem cells proliferated seven times more in space in comparison to ground based controls [54]. However, further research is needed to determine if the ocular changes observed in astronauts participating in prolonged spaceflights are similar [55].

The first study that suggested that the gut microbiome may affect the distal ocular/retinal immune system was in the mouse model of autoimmune uveitis, whereby retina-specific T cells were activated by microbiota-derived antigens further triggering autoimmunity [56]. Furthermore, another recent study demonstrated that microbiota from obese mice can activate the retinal innate immune system leading to pathological angiogenesis, through the production of pro-inflammatory mediators [13,57]. This study depicted that a high-fat diet coupled with weight gain made mice more susceptible to experimental laser-induced choroidal neovascularization. It is worth noting that when animals fed on the high fat diet were administered an oral non-gut permeable antibiotic, choroidal neovascularization was prevented, indicating the connection between the gut microbiome and ocular angiogenesis [13,52]. Moreover, when experiments of transplantation of fecal microbiota from healthy mice to obese mice by oral gavage were performed, it was found that pathological angiogenesis was markedly reduced, indicating that gut microbiome dysbiosis was a key player in this process [13,52]. The study also reported that a diet high in fat increased gut permeability, and this triggered the release of potent pro-inflammatory mediators comprising interleukin-1b (IL-1β), interleukin-6 (IL-6) and tumor necrosis factor (TNF), which were detected in the ocular choroid, as well as the pro-angiogenic vascular endothelial growth factor a (VEGFa) [13,52]. This study provided an innovative connection between the gut microbiota and ocular angiogenesis that is mediated via systemic and local immune processes. Given that several studies document a wide range of neuro-ophthalmic alterations in astronauts during and after spaceflight, namely the condition SANS, studying the connection between the gut microbiome and visual impairment in astronauts is warranted. Investigations into whether dietary interventions can help control the gut dysbiosis observed in astronauts in a microgravity environment and the effects on angiogenesis, which may lead to visual impairment needs, are required. In fact, modulating the gut microbiome might be a likely novel therapeutic option for astronauts as well the incorporation of fiber-rich prebiotics or specifically formulated probiotics.

## 5. Angiogenesis and Skeletal Muscle

**Figure 1:**
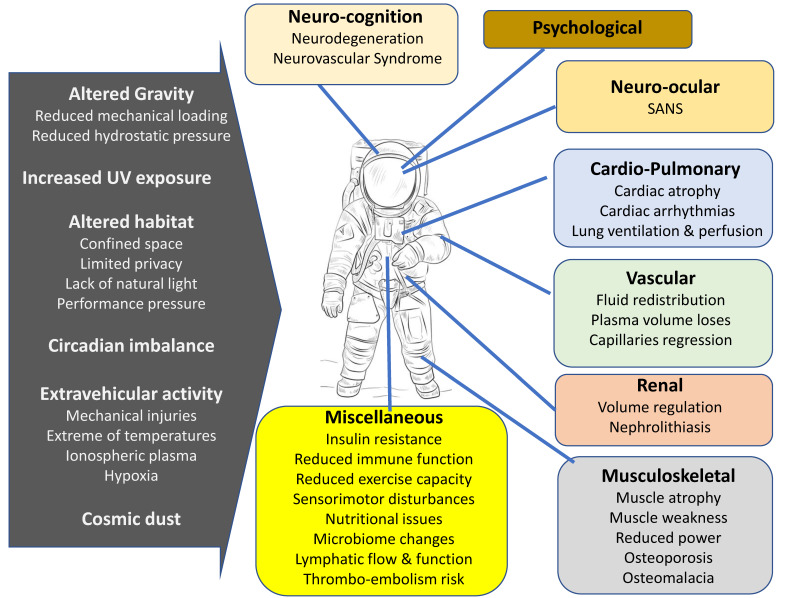
Hazards associated with spaceflight and their effects on various body systems. Spaceflight induces multiple alterations in neurological, cardiac, vascular, musculoskeletal and other systems in human body.

A noteworthy effect of long-term stay in microgravity environments on astronauts is the observation of muscle atrophy and bone loss occurring in weight-bearing bones and skeletal muscles. While mechanical unloading is the primary driver of musculoskeletal loss in space, several factors and physiological changes can potentially contribute to bone and muscle loss in space (Figure 1) [58].

Skeletal muscle is the largest organ in the body and comprises around 40% of the body mass. Traditionally studied in the context of locomotion, skeletal muscle also has a specialized metabolic function and is responsible for the storage of glucose and fats. Thus, in conditions such as hyperinsulinemia and exercise [59,60], it is one of the primary organs involved in the storage and breakdown of glucose. It is also a highly plastic organ and can adapt to altered structural and functional demands in several physiological and pathological conditions [61]. The muscle adaptation process includes alterations in muscle size and strength, fiber-type transformation as well as metabolic and contractile properties. The muscle microcirculation is an important interface for the exchange of gases, nutrition and metabolites between skeletal muscle and systemic circulation. A surfeit of evidence suggests that the microcirculation alters in parallel with the skeletal muscle in various physiological and pathological conditions [19]. This adaptation involves formation or regression of capillaries according to altered metabolic demand of the skeletal muscle. Over the years, significant advancements have been made in our understanding of the molecular mechanisms driving the alterations in muscle capillaries in various physiological and pathological conditions. Several signature molecular candidates have been identified, which play a role in muscle capillarization. However, the molecular mechanisms associated with capillary formation in these conditions have received negligible attention. Owing to the significant role of angiogenesis in muscle adaptation and the contribution of skeletal muscle to functional and metabolic health, a detailed understanding of muscle angiogenesis in microgravity conditions is required.

The formation of new capillaries from existing blood vessels, termed angiogenesis, occurs in response to exercise and wound healing. Two types of angiogenesis are recognized in skeletal muscle: splitting and sprouting [19]. Splitting angiogenesis occurs in response to sheer stress in the lumen of capillaries following low-intensity, high-volume exercise and may not involve proliferation of endothelial cells [62]. Sprouting angiogenesis occurs in response to mechanical stretch to blood vessels following overload exercises. The new and existing capillaries generally run parallel to the muscle fibers in a tortuous manner to increase the contact area with muscle [62]. Approximately 10–20 capillaries are connected to a terminal arteriole, which controls the perfusion of muscle through vasomotion [63]. The blood flow through the capillary bed can rise by 100-fold during intense contraction to meet the elevated oxygen demand of the muscle [64]. The distribution of capillaries on the surface of muscle fibers varies with the fiber size, with more homogenous distribution on small oxidative fibers compared with fast glycolytic fibers [62]. Owing to the high mitochondrial density in the slow oxidative fibers, the capillaries’ spacing seems to be designed to provide optimal oxygen to the working mitochondria. Evidence for this optimization of oxygen supply comes from the observations that the muscle fatigue resistance has a direct correlation with capillary density. Consistent with this, the muscle with elevated endurance capacity shows higher capillary density compared with the untrained, fatigue-sensitive muscles [65]. Muscle capillary density also dictates the capacity for glucose uptake and storage in the skeletal muscle. As stated earlier, skeletal muscle is one of the major storage organs for glucose. The elevated post-prandial insulin levels increase the blood flow to skeletal muscle, which enhances the glucose uptake to the skeletal muscle fibers [63]. Consistent with this, an elevated capillary density in skeletal muscle, as in endurance runners, is associated with insulin sensitivity and glucose tolerance [66]. Conversely, a diminished capillary density in sedentary and/or elderly humans contribute to glucose intolerance and insulin resistance [67]. Current studies suggest that gut microbiome may be an important factor involved in the regulating bone physiology [68]. The consequences of gut dysbiosis likely involve convoluted mechanisms resulting in modifications in the intestinal absorption of vitamins and minerals, and the regulation of inflammation as well as immunity. Recently, it was depicted that inflammation due to gut microbiome and its metabolites is mediated by the production of TNFα and the osteoclastogenic factor RANKL (receptor activator of nuclear factor kappa-B ligand) in bone, resulting in bone loss. Furthermore, the effect of the gut metabolites on bone depends on bacterial peptidoglycan sensing by the NOD receptors. Some in vivo studies in mice have reported a potential association between skeletal muscle mass and strength and *Lactobacillus* species, and further experiments, particularly in astronauts, are necessitated [69].

## 6. Metabolite Drivers of Angiogenesis and the Gut Microbiome

Studies to understand the molecular research to unravel the molecular foundations of angiogenesis commenced in the early 1970s. Recently, in the past decade or so, there has been an increase in studies on endothelial cell metabolism. These studies have provided an understanding of the role of several metabolic pathways and enzymes in angiogenesis [70]. The studies indicated that pro-angiogenic molecules, such as vascular endothelial growth factor (VEGF) and fibroblast growth factor (FGF2), as well as other growth factors are reliant on downstream metabolic adaptations for the induction of vessel sprouting, and numerous studies indicate the possibility of targeting endothelial cell metabolic pathways [71]. Recently, a transcriptome analysis of cultured endothelial cells, isolated from human fetal organs (heart, liver, lung and kidney), depicted that endothelial cells reveal the highest rates of oxygen consumption and glycolysis and are supported by upregulated genes that are linked with metabolic regulation, thus suggesting that metabolites might be involved in the regulation of endothelial differentiation into organ-specific vasculature [72].

In another recent study, metabolites released from hypoxic tissues were observed to be angiogenic; however, their role in the upregulation of genes such as vascular endothelial growth factor (VEGF) are not yet understood. In this study, various metabolites and their role in angiogenesis were determined, namely, lactate, malate, pyruvate and adenosine were investigated in vitro in human umbilical vein endothelial cells (HUVECs) that were cultured with fibroblasts of dermal origin. In addition, ethanol was tested. This study revealed that ethanol metabolism resulted in higher levels of malate and lactate. Overall, the study revealed that malate, lactate, ethanol and adenosine generated a marked angiogenic response at certain concentrations. Moreover, this angiogenic response was abolished in the presence of neutralizing anti-VEGF antibodies. The data of this study indicated that the angiogenic role of metabolites is related with increased expression of VEGF [73].

**Figure 2:**
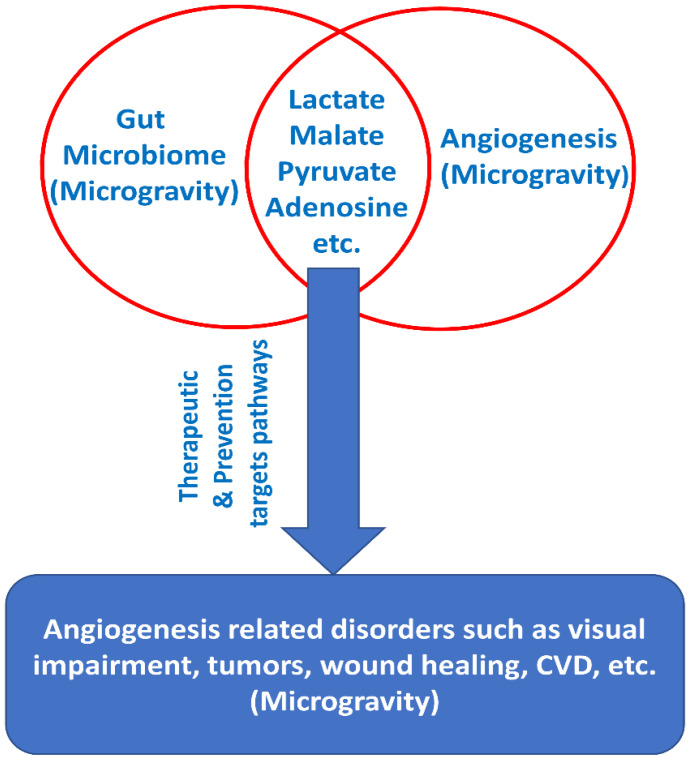
Gut microbiome and angiogenesis under microgravity environment exhibit several common metabolites including lactate, malate, pyruvate and adenosine, which could lead to the rational development of preventative measures.

Gut microbiome metabolites are essential in maintaining intestinal immune homeostasis and are biosynthesized by the gut microbiota and are involved in the regulation of intestinal epithelial cells. Short-chain fatty acids, such as acetate, propionate and butyrate, derived from bacterial anaerobic fermentation of complex polysaccharides, as well as anaerobic fungi in the colon, within dietary fiber, are one of the most studied metabolites [74,75]. Interestingly, the MARS500 study and the NASA twins study revealed that the butyrate-producing members of the gut microbiota, namely *F. prausnitzii*, were observed to alter and became depleted in all the crew members, thus indicating the importance of these bacteria and their metabolites [20]. *F. prausnitzii* are bacteria that consume acetate and produce butyrate; they display anti-inflammatory effects by producing metabolites such as butyrate, which has an anti-inflammatory role within the gut environment [76,77]. As stated above, these metabolites are also involved in angiogenesis [73]. Furthermore, studies have reported that short-chain fatty acids impact various gut immune cells to inhibit inflammation through various mechanisms [78]. Recent research only elucidates the influence of the gut microbial metabolites and how their impact extends beyond the gastrointestinal tract, such as the gut–brain axis [79]. Studies to determine if the metabolites described above and which other gut microbiome metabolites are linked to angiogenesis and microgravity environments need to be conducted in order to understand the effects on astronaut health, as well as allowing for the modulation of astronaut gut microbiome accordingly (Figure 2).

## 7. Utilization of Pro-, Pre- and Post-Biotics

As it is evident, the gut microbiome impacts the health of the host in a significant manner. The addition of probiotics added to the diet or as supplements during spaceflights is promising for improving the dysregulation faced by astronauts, which could be caused by dysbiosis in their gut and/or affect angiogenesis. In a recent study, the effectiveness of pro- or pre-biotics was determined under microgravity conditions [80]. The efficacy of the probiotic strain *L. acidophilus* microgravity environments was elucidated, and it was found that there were no obvious differences in the survival and growth, suggesting that this strain should be able to maintain its effects during space travel [80]. This was followed up by another study whereby a probiotic that was freeze-dried was developed for astronauts. The probiotic was tested for stability over a period of storage of 1 month on board the ISS. The results revealed that the probiotic has similar viable cells in comparison to controls on the ground, thus being indicative of its potential use during space travel [81]. These experiments are very encouraging; nonetheless, additional mechanistic studies under microgravity and simulated space environment are necessary, particularly to directly test the benefits of probiotics in space. In addition, postbiotics or simply metabolites secreted or released by gut bacteria are beneficial to the host [82]. Moreover, the addition of short-chain fatty acids could be of potential, as the MARS500 study revealed alteration in these metabolites. A noteworthy study known as the “the Astronaut Microbiome project” depicted a decrease in *Akkermansia*, which is associated with anti-inflammatory properties; therefore, prebiotics containing *Akkermansia* may be utilized in future work in order to prevent disease due to inflammation [12,20]. As and when the bacterial strains that are beneficial are identified and selected, intervention studies in space travelers and clinical trials are warranted in order to validate their potential. Moreover, further studies are needed to identify additional probiotics, in particular, targeting angiogenesis to maintain skeletal muscle and bone density, as well as preventing visual impairment amongst astronauts and their overall health and wellbeing.

## 8. Conclusions

The crucial role of the gut microbiome is evident in regulating the overall health of the host; likewise, the gut microbiome plays a significant role in modulating the health of astronauts during space travel, as evidenced by numerous studies. The gut microbiome modulates the immune system, metabolic health, neurological health as well as maintaining the physiology of muscles and bones and has been shown to affect distal ocular/retinal immune system, all issues that potentially affect astronauts during spaceflight. Thus, regulation of the gut microbiome is of utmost importance in the success and planning of long-term missions to space. It will be necessary to optimize the diets of astronauts, in particular, to provide dietary fiber and allow for short-chain fatty acid production, concomitantly with prebiotics, bioactive compounds and probiotics for enhanced effects. Another option to consider is transplantation from fecal microbiota in astronauts. These could be prepared from the crew members’ stool into probiotic capsules prior to travel when their microbiome is healthy on Earth. This could potentially allow astronauts with the ability to diversify their microbiome with their own diverse set of microorganisms [28]. In addition, research to explore the effects of angiogenesis and the link with the gut microbiome under the microgravity environment as well as simulated microgravity environments is needed, coupled with clinical studies on astronauts, as most of the current studies are in vitro. Future studies, in particular, for the production of short-chain fatty acids, which were observed to be altered in the MARS500 study, as well as other gut metabolites that affect angiogenesis are warranted, and will be critical in ensuring the success of future space missions.

## Data Availability

The data presented in this study are available on request from the corresponding author.
